# Bone mineral density and bone microarchitecture in a cohort of patients with Erdheim-Chester Disease

**DOI:** 10.1186/s13023-020-01518-1

**Published:** 2020-09-04

**Authors:** Tianhua He, Lijia Cui, Na Niu, Fengdan Wang, Huilei Miao, Hao Zhao, Xuemin Gao, Chang Liu, Fan Yu, Yan Jiang, Ou Wang, Mei Li, Xiaoping Xing, Daobin Zhou, Jian Li, Xinxin Cao, Weibo Xia

**Affiliations:** 1 Department of Hematology, Peking Union Medical College Hospital, Chinese Academy of Medical Sciences, Beijing, 100730 China; 2 Department of Endocrinology, Key Laboratory of Endocrinology, Ministry of Health, Peking Union Medical College Hospital, Chinese Academy of Medical Sciences, Beijing, 100730 China; 3 Department of Nuclear Medicine, Peking Union Medical College Hospital, Chinese Academy of Medical Sciences, Beijing, 100730 China; 4 Department of Radiology, Peking Union Medical College Hospital, Chinese Academy of Medical Sciences, Beijing, 100730 China

**Keywords:** Non-Langerhans histiocytosis, HR-pQCT, Chinese

## Abstract

**Background:**

Erdheim-Chester Disease (ECD) is a rare type of non-Langerhans histiocytosis. Skeletal structures are affected in over 95% ECD patients. Due to the lack of proper imaging assessment tools, the alteration of bone microarchitecture in ECD has not been well studied. High-resolution peripheral quantitative computed tomography (HR-pQCT) is a newly developed assessment of bone mineral density and bone microarchitecture.

**Methods:**

We performed a cross-sectional study with 13 patients diagnosed with ECD in Peking Union Medical College Hospital between October 2018 and June 2019. The diagnosis of ECD was based on typical pathological findings in the context of appropriate clinical and radiological manifestations. Bone geometry, volumetric bone mineral density and bone microarchitecture of those ECD patients were assessed using HR-pQCT at the non-dominant distal radius and distal tibia. Those HR-pQCT parameters were then compared to an ongoing population-based database of HR-pQCT for Mainland Chinese.

**Results:**

As a result, remarkable heterogeneity of osteosclerosis in the HR-pQCT images was found in ECD patients, ranging from apparent normal structure, scattered thickening of trabecula, to homogenous consolidation. In terms of quantitative measurements, total volumetric BMD (383.50 mg/cm^3^, 1.352 times of normal mean, *p* = 0.023) of the tibia differed significantly in ECD patients, due to the increased trabecular volumetric BMD (291 mg/cm^3^, 2.058 times of normal mean, *p* = 0.003). The increased trabecular volumetric BMD of tibia was associated with remarkably increased number of trabecula (1.7/mm, 1.455 times of normal mean, *p* = 0.002) and increased thickness of trabecula (0.37 mm, 1.466 times of normal mean, *p* = 0.003). These differences could be due to the existence of dense bone interposed in the trabecula.

**Conclusion:**

This study is the first to assess the volumetric bone mineral density and bone microstructure with HR-pQCT in a cohort of ECD patients and indicated that the application of HR-pQCT may help to reveal the nature of bone lesions in the disease.

## Background

Erdheim-Chester Disease (ECD) is a rare type of non-Langerhans histiocytosis, characterized by multi-system infiltration with lipid-laden foamy histiocytes positive of CD68 and negative of CD1a [1, 2]. Over 1500 cases of ECD have been reported worldwide [3]. Skeletal structures are affected in over 95% ECD patients [4], while extra-osseous lesions have also been reported, including of the cardiovascular system, retroperitoneum, central nervous system (CNS), and skin involvement. Typical skeletal manifestation of ECD is bilateral osteosclerosis in the metaphyseal and diaphyseal regions [4]. Several imaging techniques have been used to evaluate the bone involvement of ECD. Bone scintigraphy of ECD patients may show pathological uptake in long bones due to cortical osteosclerosis [2]; positron emission tomography-computed tomography (PET-CT) has advantages in showing the extent and activity of ECD lesions, and the therapeutic response to BRAF inhibitor, MEK inhibitor and interferon-α [3, 5–9]; computed tomography (CT) and magnetic resonance imaging (MRI) may help image-guided biopsy for ECD diagnosis [1]. However, due to the lack of proper imaging assessment tools, the alteration of bone microarchitecture in ECD has not been well studied.

High-resolution peripheral quantitative computed tomography (HR-pQCT) is a recently developed three-dimensional imaging tool that assesses volumetric bone mineral density (vBMD) and bone microarchitecture in vivo in peripheral skeletal sites [10]. HR-pQCT works by scanning the peripheral bones (mainly tibia and radius), automatically recognizing and separating the bone cortex and trabecula, obtaining multiple bone microarchitecture parameters by direct measurement and mathematical modeling, and reconstructing three-dimensional skeletal images for visualization [11]. HR-pQCT is superior to conventional dual-energy X-ray absorptiometry (DXA) in the following aspects: (1) HR-pQCT examines the three-dimensional volumetric bone mineral density (BMD) while DXA assesses the two-dimensional areal BM; (2) HR-pQCT provides a higher resolution to differentiate between bone cortex and trabecula than DXA; (3) HR-pQCT assesses bone microarchitecture in a clear view. In recent years, HR-pQCT has already been applied in examining the bone quality in metabolic bone diseases including osteoporosis [12, 13], hereditary hypophosphatemic osteomalacia [14], vitamin-D dependent rickets [11], and hematological diseases including monoclonal gammopathy of undetermined significance (MGUS) [15]. The skeletal involvement in ECD is severe and common, and the nature of bone lesions in the disease remains unknown. Due to the rarity of this disease, HR-pQCT has never been applied in ECD before. In this study, we performed a cross-sectional study of HR-pQCT in 13 ECD patients in our center to examine their changes in vBMD and bone microarchitecture.

## Methods

### Patients enrolled

Thirteen patients diagnosed with ECD in Peking Union Medical College Hospital (PUMCH, Beijing, China) were enrolled in the study between October 2018 and June 2019. The diagnosis of ECD was based on typical pathological findings in the context of appropriate clinical and radiological manifestations [4]. Patients were excluded if they were also diagnosed with autoimmune diseases, osteoporosis, other osteosclerotic diseases, other malignancies, bone fractures of radius or tibia, or have been treated with glucocorticoids in the past 1 year.

Demographic characteristics and clinical manifestations were documented at the time of enrollment, and HR-pQCT was completed within 2 weeks. For newly diagnosed patients, HR-pQCT was done before treatment was initiated. Treatment-experienced patients were treated with high-dose interferon-α therapy, defined as 600 or 900MIU three times per week.

Ethics approval was obtained for the study from the institutional board. All participants were informed both in person by TH, and written informed consents were obtained. The study was performed in accordance with the ethical standards of the 1964 Declaration of Helsinki and its later amendments.

### Evaluation and definition

Multisystem involvement was confirmed by either clinical symptoms or radiological findings. Cardiovascular system involvements, valve abnormalities or periaortic fibrosis, were evaluated with cardiac MRI or CT, or echocardiograph. CT scans of the chest and abdomen were also applied for assessing retroperitoneal tissue fibrosis (“hairy kidney”) and pulmonary involvement. For central nervous system involvement, we introduced MRI for patients with related symptoms. PET-CT has also been performed for evaluating whole-body multisystem involvement.

### High-resolution peripheral quantitative computed tomography (HR-pQCT)

Bone geometry, volumetric bone mineral density (vBMD) and bone microarchitecture were assessed using HR-pQCT (XtremeCT II; Scanco Medical, Zurich, Switzerland) at the non-dominant distal radius and distal tibia. Each measurement was initiated at 9.5 mm and 22.5 mm from the mid-endplate at the radius and tibia, respectively. One hundred sixty-eight parallel slices were obtained in the axial direction, providing images with an isotopic image voxel size of 82 μm. The image quality was assessed by an experienced technician using the 5-step scale, and the images with quality worse than grade 3 were excluded from the analysis [16].

The contour between cortex and trabecula was defined automatically using the manufacturer’s standard software with manually assistance by one of the authors (TH). The following measurements were calculated: 1) Bone geometrical measures including total (Tt.Ar), cortical (Ct.Ar) and trabecular (Tb.Ar) areas (mm^2^), 2) vBMD (mg hydroxyapatite/cm^3^) of the entire cross section (Tt.vBMD), cortical section (Ct.vBMD) and trabecular section (Tb.vBMD), and 3) microarchitectural parameters including trabecular bone volume to tissue volume fraction (BV/TV), trabecular number (Tb.N, mm-1), trabecular thickness (Tb.Th, mm), trabecular separation (Tb.Sp, mm), standard deviation of 1/Tb.N to represent trabecular network inhomogeneity (Tb.1/N.SD, mm), cortical thickness (Cr.Th, mm) and cortical porosity (Ct.Po, %).

### Other bone imaging

All patients had the ^18^F-Fluorodeoxyglucose (FDG) positron emission tomography (PET) scan and 99 m methylene diphosphonate (^99m^Tc-MDP) bone scintigraphy for a better definition of the range of bone lesions. We also documented X-rays of affected regions as additional evaluations. One patient has also taken MRI of the affected ankle to determine the nature of bone lesions.

### Data processing and statistics

All statistical analyses were performed using R (R version 3.6.0, 2019-04-26, 2019 The R Foundation for Statistical Computing Platform) [17]. Age-, gender- and site specific distributions were derived from generalized additive models for location, scale, and shape (GAMLSS) with age as the only explanatory variable, based on an ongoing population-based study of HR-pQCT for Mainland Chinese [18, 19] (Supplementary Table 1). For each patient, Z-scores of each measurement were derived from the fitted distribution models, as well as the reference mean of the healthy population. All measurements of HR-pQCT were compared with and adjusted to the corresponding reference normal mean. One sample *t*-test was performed comparing each relative value to 1, and *p* value < 0.05 was considered to be statistically significant.

## Results

### Patients

Thirteen ECD patients were enrolled in the study (Table 1), with a median age of 47 years (range 19–62) and an even distribution of males and females. One patient was ECD overlapped with Langerhans cell histiocytosis (LCH). Seven were treatment-naïve patients, of which one patient had repeated HR-pQCT assessment after a 6-month treatment. The other treatment experienced patients had intermittent high-dose interferon injection for 15–46 months. *BRAF* V600E mutation was positive in five out of nine patients tested by either PCR or immunohistochemical approach.

**Table 1:** Demographic features of 13 ECD patients

ID	Sex/ Age	Organ involvement	Bone scintigraphy	Biopsy position	BRAF-PCR/immuno- histochemistry	Disease duration (months)	Treatment history	Regimen/Treatment duration (months)
LJ004	M/46	Bone, heart, vasculature, retroperitoneal, peritoneum	Typical uptake	Diaphragm	(−)/NA	19	No	\
LJ008	F/49	Bone, CNS, heart, vasculature	Typical uptake	Foramen magnum	NA/NA	48	No	\
LJ013	F/30	Bone, CNS, pancreas	Typical uptake	Sellar region	NA/(−)	74	No	\
LJ019	M/19	Bone, lungs, retroperitoneal, pleura, exophthalmos	Typical uptake	Right lung	(+)/NA	22	Yes	IFN/9
LJ020	M/54	Bone, exophthalmos	Typical uptake	Periorbital region	NA/NA	39	No	\
LJ023	F/57	Bone, lungs	Typical uptake	Tibia	(+)/(+)	63	Yes	IFN/15
LJ024	F/26	Bone, CNS, thymus, exophthalmos	Typical uptake	Periorbital region	(+)/(+)	246	Yes	IFN/24
LJ025	F/53	Bone, exophthalmos	Typical uptake	Periorbital region	NA/NA	49	No	\
LJ033	M/49	Bone, pleura, nerve roots	Typical uptake	Pleura, intraspinal mass	(+)/NA	71	Yes	IFN/46
LJ038	M/62	Bone, vasculature, retroperitoneal, pericardium	Typical uptake	Pericardium	(+)/(+)	34	Yes	IFN/30
LJ043	F/47	Bone, vasculature, retroperitoneal, exophthalmos	Typical uptake	Periorbital region	NA/(−)	138	No	\
LJ045	M	Bone, CNS, lungs, vasculature, gingiva	Typical uptake	Gingiva	(−)/NA	255	Yes	IFN/35
LJ047	M	Bone	High uptake within Distal right fibula	Right fibula	NA/NA	4	No	\

All patients enrolled had bone involvement confirmed by PET-CT or bone scintigraphy, and the sites of bone lesions were listed in Table 1.

### Geometry, vBMD, and microarchitecture by HR-pQCT

Bone geometry, volumetric BMD, microarchitecture measurements obtained by HR-pQCT were shown in Table 2 and Supplementary Table 2. Tt.vBMD (383.50 mg/cm^3^, 1.352 times of normal mean, *p* = 0.023) of the tibia differed significantly in ECD patients, attributing to the increased Tb.vBMD (291 mg/cm^3^, 2.058 times of normal mean, *p* = 0.003). The increased Tb.vBMD of tibia was associated with remarkably increased number of trabecula (Tb.N, 1.7/mm, 1.455 times of normal mean, *p* = 0.002) and increased thickness of trabecula (Tb.Th, 0.37 mm, 1.466 times of normal mean, p = 0.003). There were no significant differences in geometric features (Tt.Ar, Ct.Ar, or Tb.Ar) or microachitectural features in the cortex of tibia (Ct.Th or Ct.Po). And most HR-pQCT parameters in the radius showed no significant difference.

**Table 2:** HR-pQCT measurements in 13 ECD patients

	Tibia			Radius		
	Mean ± standard deviation	Mean of adjusted value*	*P*-value**	Mean ± standard deviation	Mean of adjusted value*	P-value**
*Bone geometry*						
Total area (mm2)	722.39 ± 81.79	1.003 ± 0.104	0.924	286.64 ± 61.63	0.992 ± 0.170	0.859
Cortical area (mm2)	115.01 ± 36.79	0.887 ± 0.363	0.273	60.75 ± 11.72	0.871 ± 0.204	0.032
Trabecular area (mm2)	612.07 ± 81.88	1.029 ± 0.104	0.320	229.63 ± 58.64	1.029 ± 0.199	0.599
*Bone mineral density*						
Total BMD (mg/cm3)	383.5 ± 127.69	1.352 ± 0.521	0.023	367.96 ± 120.92	1.097 ± 0.399	0.391
Trabecular BMD (mg/cm3)	291.87 ± 146.46	2.058 ± 1.165	0.003	223.15 ± 150.06	1.529 ± 1.246	0.139
Cortical BMD (mg/cm3)	886.37 ± 83.37	0.952 ± 0.085	0.052	887.94 ± 76.93	0.955 ± 0.080	0.055
*Microarchitecture measurements*						
Trabecular BV/TV	0.41 ± 0.2	1.876 ± 1.005	0.004	0.33 ± 0.23	1.570 ± 1.307	0.129
Trabecular number (1/mm)	1.7 ± 0.53	1.455 ± 0.469	0.002	1.58 ± 0.54	1.154 ± 0.392	0.170
Trabecular thickness (mm)	0.37 ± 0.12	1.466 ± 0.509	0.003	0.31 ± 0.17	1.361 ± 0.783	0.110
Trabecular spacing (mm)	0.60 ± 0.20	0.865 ± 0.293	0.109	0.68 ± 0.36	0.977 ± 0.464	0.858
Standard deviation of 1/trabecular number	0.25 ± 0.09	0.763 ± 0.274	0.005	0.30 ± 0.22	1.124 ± 0.721	0.540
Cortical thickness (mm)	1.30 ± 0.42	0.909 ± 0.343	0.348	1.03 ± 0.20	0.887 ± 0.189	0.041
Cortical porosity (%)	0.02 ± 0.02	1.346 ± 0.904	0.180	0.01 ± 0.01	1.854 ± 2.312	0.196

^a^ The adjusted value was the quotient of the measurements and that of the reference normal mean, which derived from an ongoing population-based study of HR-pQCT in Chinese population [19]. Details of fitted distribution model was listed in supplementary document

^b^ p-value: one sample t-test

Abbreviations: BMD, bone mineral density; BV/TV, bone volume to tissue volume fraction;

### Patterns of bone lesions by HR-pQCT image

**Fig. 1:**
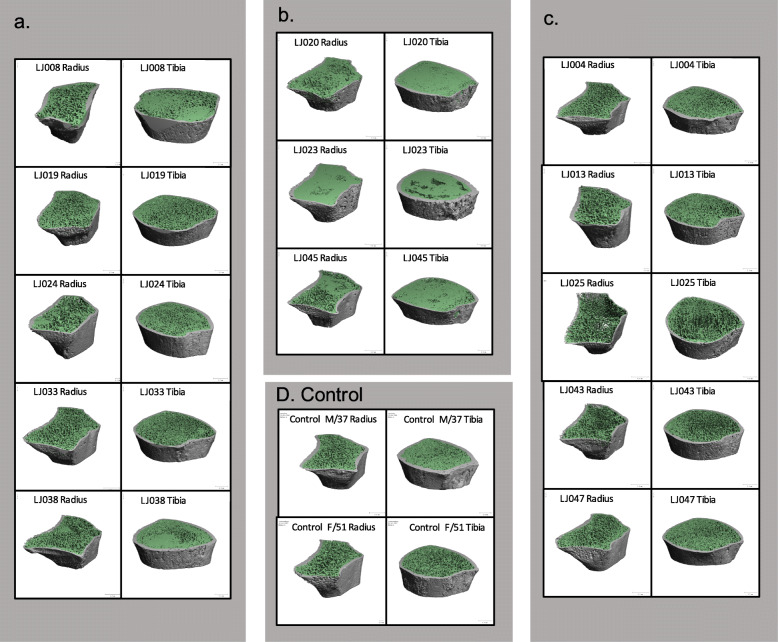
HR-pQCT three-dimensional images of 13 ECD patients at distal radius (R) and tibia (T), grouped by their bone microarchitecture features: **a** scattered trabecular thickness, **b** homogeneously osteosclerosis, or **c** apparently normal structures. **d** HR-pQCT three-dimensional images of healthy controls

As shown in Fig. 1, great heterogeneity in bone microarchitecture was found in HR-pQCT images in ECD patients. Some ECD patients presented scattered thickening of trabecula (LJ008, LJ019, LJ024, LJ033 and LJ038), some presented homogeneous osteosclerosis (LJ020, LJ023 and LJ045), and others showed apparently normal structure. Besides, tibia was involved in most ECD patients (Fig. 1) with more evident distortion of bone structures, while radius was involved in less of them.

### Comparison of HR-pQCT with other bone imaging

**Fig. 2:**
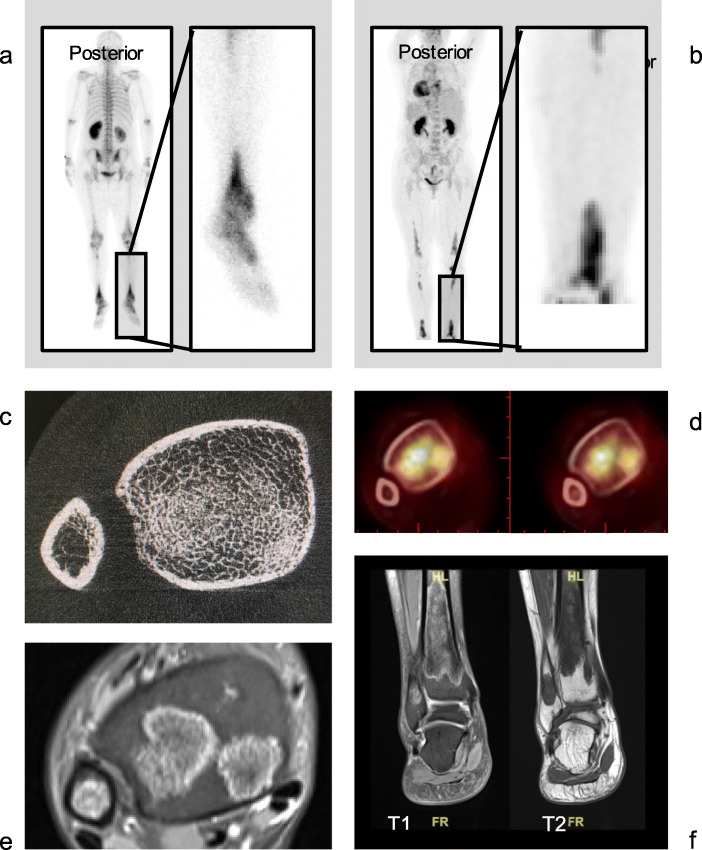
Images of bone scintigraphy, positron emission tomography-computed tomography (PET-CT), HR-pQCT and Magnetic Resonance Imaging. (MRI) in one representative treatment-naïve ECD patients (LJ008). **a** Bone scintigraphy showed bilateral symmetric uptake at the distal tibial region. **b** PET-CT showed abnormal uptake at the same tibial region. **c** HR-pQCT revealed localized structural alteration of trabeculae network in right tibia. **d** Cross-sectional view of PET-CT showed increase of uptake in tibia, where bone lesions existed, reaching a SUV maximum of 5. **e** and **f** MRI of affected left ankle showed localized heterogeneous low T1- and T2-signal, resembling that of cortex

Figure 2 presented the bone lesions of one representative patient (LJ008) evaluated by bone scintigraphy, MRI, HR-pQCT, and PET-CT. Bone scintigraphy and PET-CT demonstrated characteristic bilateral distal tibia osteosclerosis (Fig. 2A and B). The MRI of left ankle revealed a localized lesion, with heterogenic low signal on T1- and T2-weighted image, resembling corticalized loci within the trabecular regions (Fig. 2F). Comparing MRI cross-sectional image (Fig. 2E) with HR-pQCT (Fig. 2C), those typical bone lesions showed an infiltration of dense mass with the original trabecular networking spared.

### HR-pQCT measurements before and after treatment

HR-pQCT was repeated in one ECD patient (LJ004) before and after interferon treatment for 6 months. As shown in Table 3, no significant changes in volumetric BMD and microarchitecture measurements were found before and after treatment.

**Table 3:** Compare HR-pQCT measurements pre- and six-month post- treatment of one representative patient (LJ004)

	Pre-Tx Tibia	Post-Tx Tibia	Pre-Tx Radius	Post-Tx Radius
**Geometric features**				
Total area (mm2)	784.8	776.4	335.8	301.4
Cortical area (mm2)	147.2	146.2	72.9	76.1
Trabecular area (mm2)	643.3	635.9	267.1	229.1
**Volumetric BMD**				
Total BMD (mg/cm3)	350.7	361.1	310.1	337.7
Trabecular BMD (mg/cm3)	223.7	228.5	145	134.8
Cortical BMD (mg/cm3)	911.3	944.8	930.2	960.6
**Microarchitecture measurements**				
Trabecular BV/TV	0.326	0.328	0.204	0.189
Trabecular number (1/mm)	1.885	1.462	1.467	1.516
Trabecular thickness (mm)	0.305	0.322	0.216	0.216
Trabecular spacing (mm)	0.536	0.696	0.647	0.637
Standard deviation of 1/trabecular number	0.22	0.293	0.228	0.23
Cortical thickness (mm)	1.58	1.616	1.123	1.247
Cortical porosity (%)	0.01	0.01	0.007	0.004

Abbreviations: BMD, bone mineral density; BV/TV, bone volume to tissue volume fraction;

## Discussion

This study is the first to assess the bone microstructure with HR-pQCT in a cohort of patients diagnosed with ECD. Our data revealed great heterogeneity in bone microarchitecture alteration within trabecular region, from apparent normal structure, scattered thickening of trabecula, to homogeneous consolidation of trabecula. In terms of quantitative measurements of bone geometry, vBMD, and microarchitecture, ECD patients had significantly increased Tb.vBMD, Tb.Th, Tb.N, and BV/TV in the distal tibia, which could be due to the existence of dense bone interposed in the trabecula.

Though the etiology of ECD remains unclear, ECD is now believed to be a clonal disorder marked by frequent hyper-activation of mitogen-activated protein kinase signaling [20]. The mixed distribution of dense and normal bones may be attributed to the active bone resorption and its secondary osteogenesis, due to an altered inflammatory milieu, as shown in Fig. 2E and F. MRI further suggested bone infarction with secondary osteogenesis, showing serpiginous peripheral low T1 and high T2 signal due to granulation or sclerosis, and central high T1 and low T2 signal of bone marrow. The occurrence of bone infarction can be attributed to histiocyte infiltration and alteration of microenvironment within bone marrow. This can be a slow process with necrosis, resorption, formation and ossification happening simultaneously, which partially explained why the osteosclerosis extent shown in pQCT were not strictly associated with the duration of disease (Fig. 1) nor with the duration of treatment (Table 3). Previous reported ECD cases with bone biopsy further confirmed thickened trabeculae with osteosclerosis and increased bone marrow cellularity, which was related to infiltration of foamy histiocytes [21]. Another review of pathological findings of ECD affected bones also found replacement of normal bone marrow with fibrosis and sclerosis of varied degree, and reactive bone formation was commonly seen [22]. Further studies with long-term follow-up and repeated evaluation of the morphology evolutions by MRI, microarchitecture alterations by HR-pQCT, and metabolic activities by bone scintigraphy and PET/CT, may help in understanding the nature of bone lesions in ECD.

We found different patterns of bone microarchitecture alteration among ECD patients, and such changes were predominantly within trabecular regions, while cortical regions remain spared. Our 3D microarchitecture helped to differentiate the thickened marginal trabeculae with the cortex, especially for patients with acentric and multiple lesions. Therefore, classically known bone lesions of ECD with cortical thickness and sclerosis may result from insufficient resolution and misread. Our findings from HR-pQCT was consistent with pathological findings of replacement of normal bone marrow with fibrosis and sclerosis [22].

As the clinical spectrum of ECD ranges from asymptomatic to multi-organ lesions, we think that the different patterns and extent of bone microarchitecture alteration could be attributed to the different stages of the disease. Dion and colleagues have reported similar findings in 11 ECD patients based on X-ray, as 65% of the patients presented heterogeneous osteosclerosis and 35% of them presented homogenous osteosclerosis [23].

The phenomenon of scattered thickening of trabeculae is very rarely seen in other bone diseases. A similar pattern has only been reported in autosomal dominant osteopetrosis (ADO), which was explained as a random distribution of old and fragile bones along the skeleton by Arruda and colleagues [24]. As for osteoid osteoma, a benign tumor with featured bone formation, Rolvein et al. applied HR-pQCT to image a few cases with intra-articular osteoid osteoma and found typical subchondral nidus with central calcification and surrounding reactive bone formation [25]. Osteosarcoma is characterized by direct formation of immature bone by the tumor cells. HR-pQCT scan of murine models with osteosarcoma showed lytic intra-osseous lesions and periosteal reactive bone formation with deteriorated organization and directionality [26]. POEMS syndrome patients commonly present localized sclerotic bone lesions. We have conducted HR-pQCT for patients with newly diagnosed POEMS syndrome, and found decreased vBMD in the cortex and normal vBMD in the trabeculae, resulting from the combined effects of increased trabecular number and decreased trabecular thickness (unpublished data).

Interestingly, the occurrence of bone lesion does not always match the clinical symptoms of bone pain. Although 8 of all 13 patients showed marked aberrant bone lesion in HR-pQCT images (Fig. 1), only two of them presented clinical bone pain (LJ023 and LJ045). This finding indicates that the involvement of the skeletal system is common but insidious in the onset of the disease. Similarly, in another ECD cohort reported by Haroche and colleagues, skeletal involvement was found in almost all 11 ECD patients but only 50% of them suffered from bone pain [27].

There are also limitations in this study. First, this is a single center study, which may limit the generalizability of our findings. Second, HR-pQCT can only image peripheral skeletal sites, and was not fit for patients with bone lesions not close enough to the end-plate of radius and tibia. Third, we only repeated HR-pQCT in one patient’s follow-up, which did not show significant changes in quantitative assessment (Table 3). A longer follow-up and repeated HR-pQCT in more ECD patients may identify the restoration of bone lesions and the effect of IFN- α on bone remodeling.

## Conclusions

In conclusion, we studied the bone microarchitecture in 13 ECD patients using HR-pQCT imaging. Trabecular number, thickness and volumetric bone mineral density were significantly higher in ECD patients. Remarkable heterogeneity in bone microarchitecture was found in ECD patients, ranging from apparent normal structure, scattered thickening of trabecula to homogenous consolidation. The application of HR-pQCT may help to reveal the bone microarchitecture of ECD, and the nature of bone lesions in the disease.

## Supplementary information

**Additional file 1.**

## Data Availability

The datasets used and/or analysed during the current study are available from the corresponding author on reasonable request.

## Abbreviations

ECD: Erdheim-Chester Disease
CNS: central nervous system
PET-CT: positron emission tomography-computed tomography
CT: computed tomography
MRI: magnetic resonance imaging
HR-pQCT: high-resolution peripheral quantitative computed tomography
vBMD: volumetric bone mineral density
DXA: dual-energy X-ray absorptiometry
MGUS: monoclonal gammopathy of undetermined significance
GAMLSS: generalized additive models for location, scale, and shape
LCH: Langerhans cell histiocytosis
ADO: autosomal dominant osteopetrosis
