# Supplementary material for: Bone mineral density and bone microarchitecture in a cohort of patients with Erdheim-Chester Disease
Source: Orphanet J Rare Dis. 2020 Sep 4;15:236. doi: 10.1186/s13023-020-01518-1 (PMC7487812; doi:10.1186/s13023-020-01518-1)
Supplement: Supplementary file 1 — Additional file 1. [file 13023_2020_1518_MOESM1_ESM.docx]

**Supplementary document**

**Supplementary Table 1. Fitted distributions and parameters.^(1)^**

| HR-pQCT measurements | Distributions | Age^λ^ | df(μ) | df(σ) | df(ν) | df(τ) |
| --- | --- | --- | --- | --- | --- | --- |
| **Male. Radius** |  |  |  |  |  |  |
| Tt.vBMD (mgHA/cm3) | Normal | 0.000 | 4.164 | 2.000 | / | / |
| Ct.vBMD (mgHA/cm3) | BCCG | 1.500 | 4.209 | 3.399 | 2.000 | / |
| Tb.vBMD (mgHA/cm3) | Normal | 0.872 | 2.000 | 2.960 | / | / |
| Tt.Ar (mm2) | BCPE | 0.000 | 2.011 | 2.001 | 3.410 | 2.000 |
| Ct.Ar (mm2) | BCCG | 1.500 | 4.454 | 2.001 | 2.243 | / |
| Tb.Ar (mm2) | BCPE | 0.000 | 2.007 | 2.001 | 2.000 | 2.000 |
| Ct.Th (mm) | BCCG | 1.500 | 3.999 | 2.001 | 2.000 | / |
| Ct.Po (%) | BCCG | 0.000 | 3.531 | 4.273 | 2.000 | / |
| Ct.Pm (mm) | BCPE | 0.000 | 2.033 | 2.001 | 2.000 | 2.000 |
| Tb.BV/TV | Normal | 0.756 | 7.543 | 3.112 | / | / |
| Tb.N (mm-1) | Normal | 1.500 | 2.007 | 2.001 | / | / |
| Tb.Th (mm) | Normal | 1.500 | 2.691 | 2.001 | / | / |
| Tb.Sp (mm) | BCCG | 0.995 | 2.012 | 2.665 | 2.000 | / |
| Tb.1/N.SD (mm) | BCPE | 0.675 | 2.010 | 2.001 | 2.000 | 3.557 |
| **Female. Radius** |  | |  |  |  |  |
| Tt.vBMD (mgHA/cm3) | BCCG | 0.397 | 5.189 | 2.608 | 2.335 | / |
| Ct.vBMD (mgHA/cm3) | BCPE | 1.072 | 5.338 | 2.692 | 2.000 | 3.550 |
| Tb.vBMD (mgHA/cm3) | BCCG | 0.008 | 4.479 | 2.001 | 2.527 | / |
| Tt.Ar (mm2) | BCCG | 1.145 | 2.033 | 2.002 | 3.201 | / |
| Ct.Ar (mm2) | BCPE | 0.133 | 5.593 | 6.203 | 3.050 | 2.000 |
| Tb.Ar (mm2) | BCCG | 1.500 | 2.021 | 2.002 | 2.000 | / |
| Ct.Th (mm) | BCCG | 0.082 | 5.798 | 3.573 | 2.000 | / |
| Ct.Po (%) | BCPE | 2.271 | 7.500 | 2.002 | 2.000 | 2.000 |
| Ct.Pm (mm) | BCT | 1.427 | 2.105 | 2.001 | 2.851 | 2.000 |
| Tb.BV/TV | BCCG | 0.000 | 4.450 | 2.001 | 2.000 | / |
| Tb.N (mm-1) | BCCG | 0.656 | 3.825 | 2.706 | 2.000 | / |
| Tb.Th (mm) | BCCG | 0.000 | 4.724 | 4.088 | 2.000 | / |
| Tb.Sp (mm) | BCPE | 1.500 | 3.592 | 2.002 | 2.000 | 3.226 |
| Tb.1/N.SD (mm) | BCT | 1.500 | 3.205 | 2.001 | 2.000 |  |
| **Male. Tibia** |  |  |  |  |  |  |
| Tt.vBMD (mgHA/cm3) | BCPE | 0.000 | 2.030 | 4.480 | 2.000 | 4.383 |
| Ct.vBMD (mgHA/cm3) | BCPE | 0.314 | 5.743 | 2.284 | 2.000 | 3.197 |
| Tb.vBMD (mgHA/cm3) | BCPE | 0.000 | 4.033 | 4.969 | 2.000 | 2.000 |
| Tt.Ar (mm2) | BCPE | 749.000 | 2.015 | 2.001 | 2.000 | 2.000 |
| Ct.Ar (mm2) | Normal | 1.500 | 2.000 | 2.001 | / | / |
| Tb.Ar (mm2) | BCPE | 1.089 | 2.010 | 2.001 | 1.000 | 3.000 |
| Ct.Th (mm) | BCCG | 0.731 | 2.009 | 2.001 | 2.000 | / |
| Ct.Po (%) | BCPE | 0.000 | 6.531 | 2.001 | 2.000 | 2.000 |
| Ct.Pm (mm) | BCPE | 0.802 | 2.074 | 2.001 | 2.000 | 4.741 |
| Tb.BV/TV | BCPE | 0.000 | 4.248 | 6.516 | 4.635 | 2.000 |
| Tb.N (mm-1) | BCCG | 0.000 | 2.993 | 2.001 | 2.000 | / |
| Tb.Th (mm) | BCCG | 0.000 | 4.595 | 2.001 | 3.154 | / |
| Tb.Sp (mm) | BCCG | 0.000 | 2.989 | 2.001 | 2.000 | / |
| Tb.1/N.SD (mm) | BCCG | 0.000 | 2.009 | 3.587 | 3.062 | / |
| **Female. Tibia** |  |  |  |  |  |  |
| Tt.vBMD (mgHA/cm3) | BCCG | 1.500 | 2.024 | 3.130 | 2.000 | / |
| Ct.vBMD (mgHA/cm3) | BCCG | 1.140 | 7.087 | 3.388 | 2.000 | / |
| Tb.vBMD (mgHA/cm3) | Normal | 0.426 | 2.000 | 2.002 | / | / |
| Tt.Ar (mm2) | BCCG | 0.000 | 2.036 | 2.318 | 2.000 | / |
| Ct.Ar (mm2) | BCCG | 0.114 | 5.163 | 3.781 | 2.617 | / |
| Tb.Ar (mm2) | BCCG | 0.628 | 6.000 | 2.922 | 2.000 | / |
| Ct.Th (mm) | BCCG | 0.000 | 4.945 | 2.224 | 2.000 | / |
| Ct.Po (%) | BCT | 1.500 | 9.532 | 2.001 | 2.000 | 2.000 |
| Ct.Pm (mm) | BCCG | 0.000 | 2.134 | 2.518 | 2.000 | / |
| Tb.BV/TV | BCCG | 0.818 | 2.017 | 2.002 | 2.000 | / |
| Tb.N (mm-1) | BCPE | 1.500 | 2.030 | 2.208 | 3.336 | / |
| Tb.Th (mm) | BCCG | 1.500 | 2.141 | 2.002 | 3.336 | / |
| Tb.Sp (mm) | BCPE | 1.500 | 2.027 | 2.499 | 2.000 | 3.958 |
| Tb.1/N.SD (mm) | BCT | 1.500 | 2.017 | 3.849 | 2.000 | 3.419 |

Abbreviations: Tt., total; Ct., cortical; Tb., trabecular; Ar, areas; vBMD, volumetric bone mineral density; BV/TV, trabecular bone volume to tissue volume fraction; Tb.N, trabecular number; Tb.Th, trabecular thickness; Tb.Sp, trabecular separation; Tb.1/N.SD, standard deviation of 1/Tb.N; Ct.Th, cortical thickness; Ct.Po, cortical porosity; BCCG, Box-Cox Cole and Green distribution; BCPE, Box-Cox power exponential distribution; BCT, Box-Cox t distribution; Normal, normal distribution.

**Supplementary Table 2. Z-scores of HR-pQCT measurements.**

|  | sex | age | Tt.Ar | Ct.Ar | Tb.Ar | Tt.vBMD | Tb.vBMD | Ct.vBMD | Tb.BV/TV | Tb.N | Tb.Th | Tb.Sp | Tb.1/NSD | Ct.Th | Ct.Po |
| --- | --- | --- | --- | --- | --- | --- | --- | --- | --- | --- | --- | --- | --- | --- | --- |
| **Radius** |  |  |  |  |  |  |  |  |  |  |  |  |  |  |  |
| LJ004 | M | 46.1 | -0.067 | -0.902 | 0.041 | -0.623 | -0.548 | 0.273 | -0.782 | 0.196 | -1.588 | -0.104 | -0.557 | -0.600 | 0.009 |
| LJ008 | F | 48.7 | -0.429 | 1.298 | -0.845 | 1.469 | 0.900 | 1.304 | 0.870 | 1.185 | 1.065 | -1.022 | -0.996 | -0.292 | 1.791 |
| LJ013 | F | 29.8 | -0.971 | -1.858 | -0.636 | -0.903 | -0.288 | <-3 | -0.189 | -0.206 | 0.164 | 0.192 | -0.307 | >3 | -0.752 |
| LJ019 | M | 19.6 | -0.273 | -2.907 | 0.268 | 0.760 | >3 | -1.081 | >3 | 1.238 | >3 | -0.533 | -0.023 | -2.746 | 0.501 |
| LJ020 | M | 54.4 | -0.001 | -2.320 | 0.251 | 2.045 | >3 | -1.175 | >3 | >3 | >3 | <-3 | <-3 | -1.130 | -0.363 |
| LJ023 | F | 55.7 | 1.849 | 1.754 | 1.509 | >3 | >3 | <-3 | >3 | >3 | >3 | <-3 | -2.950 | 1.869 | 0.566 |
| LJ024 | F | 26.3 | -1.926 | -1.735 | -1.559 | 0.406 | -0.432 | 1.024 | -0.706 | 0.089 | 0.698 | 0.243 | 0.232 | -2.041 | -0.309 |
| LJ025 | F | 53.2 | 1.034 | <-3 | 1.484 | <-3 | -2.511 | <-3 | -2.927 | -2.618 | -1.695 | 2.450 | 2.206 | 1.210 | <-3 |
| LJ033 | M | 49.1 | 0.158 | -1.660 | 0.338 | -0.946 | -0.195 | -0.630 | -0.352 | -0.415 | -0.622 | 0.229 | 0.124 | -1.369 | 0.987 |
| LJ038 | M | 62.8 | 0.077 | -0.739 | 0.171 | 1.514 | >3 | -0.178 | >3 | 1.195 | >3 | -1.282 | -0.701 | -0.747 | -0.088 |
| LJ043 | F | 47.4 | 0.959 | 0.171 | 0.920 | -1.185 | -1.309 | -0.332 | -1.531 | -0.982 | -1.689 | 1.207 | 1.504 | 1.162 | -0.353 |
| LJ045 | M | 48.7 | -1.402 | -2.015 | -0.972 | 2.269 | >3 | 0.007 | >3 | 2.525 | >3 | -1.694 | -0.389 | -0.976 | -0.694 |
| LJ047 | M | 31.2 | -0.385 | -1.519 | -0.110 | -0.802 | -0.497 | -0.055 | -0.748 | 0.249 | -0.882 | -0.143 | -0.118 | -1.369 | -0.552 |
| **Tibia** |  |  |  |  |  |  |  |  |  |  |  |  |  |  |  |
| LJ004 | M | 46.1 | -0.183 | -0.045 | -0.182 | 0.981 | 2.049 | -0.437 | 2.123 | 2.663 | 1.918 | -2.268 | -2.113 | 0.227 | -1.499 |
| LJ008 | F | 48.7 | 0.339 | -0.362 | 0.325 | >3 | >3 | -0.201 | >3 | >3 | >3 | <-3 | -2.762 | -0.249 | -0.038 |
| LJ013 | F | 29.8 | 0.443 | -1.438 | 0.834 | -0.001 | 1.990 | <-3 | 1.387 | 1.349 | 1.787 | -1.319 | -1.385 | -1.216 | 2.083 |
| LJ019 | M | 19.6 | 0.132 | -2.320 | 0.430 | -0.985 | -0.368 | -0.554 | -0.519 | -0.573 | >3 | 0.748 | 0.116 | -2.243 | 1.973 |
| LJ020 | M | 54.4 | -0.219 | -1.863 | 0.053 | >3 | >3 | -0.694 | >3 | >3 | >3 | <-3 | <-3 | -1.594 | -1.446 |
| LJ023 | F | 55.7 | 1.416 | >3 | 0.338 | >3 | >3 | -2.265 | >3 | >3 | >3 | <-3 | <-3 | >3 | 2.148 |
| LJ024 | F | 26.3 | -1.335 | -2.347 | -0.952 | 1.304 | 2.594 | 1.534 | 2.273 | 2.252 | 2.081 | -2.432 | -2.265 | -1.384 | <-3 |
| LJ025 | F | 53.2 | 0.600 | -2.532 | 0.965 | -0.816 | 1.193 | <-3 | 0.912 | -0.458 | >3 | 0.760 | 1.329 | -2.046 | 1.015 |
| LJ033 | M | 49.1 | -0.190 | -1.739 | 0.072 | -0.084 | 1.062 | -0.109 | 0.855 | 0.745 | 1.472 | -0.838 | -0.895 | -1.655 | 0.958 |
| LJ038 | M | 62.8 | -0.811 | -0.134 | -0.749 | >3 | >3 | 0.923 | >3 | 2.723 | >3 | -2.646 | -2.275 | 0.361 | -0.028 |
| LJ043 | F | 47.4 | 0.702 | -0.022 | 0.614 | -1.080 | -1.321 | -0.470 | -1.436 | -0.017 | -0.938 | 0.307 | 0.238 | -0.288 | 0.339 |
| LJ045 | M | 48.7 | -0.040 | <-3 | 0.436 | 2.884 | >3 | -1.524 | >3 | >3 | >3 | <-3 | -2.557 | <-3 | 0.358 |
| LJ047 | M | 31.2 | -0.579 | -1.811 | -0.228 | -0.574 | -0.075 | 0.473 | -0.089 | 0.575 | -1.005 | -0.697 | -0.925 | -1.618 | -0.657 |

Abbreviations: Tt., total; Ct., cortical; Tb., trabecular; Ar, areas; vBMD, volumetric bone mineral density; BV/TV, trabecular bone volume to tissue volume fraction; Tb.N, trabecular number; Tb.Th, trabecular thickness; Tb.Sp, trabecular separation; Tb.1/N.SD, standard deviation of 1/Tb.N; Ct.Th, cortical thickness; Ct.Po, cortical porosity; M, male; F, female.

**Reference:**

1. Yu F. Age-, site- and gender-specific reference centile curves and normative data for HRpQCT-derived bone structural parameters in Chinese mainland population. Clinical Medical School: Peking Union Medical College; 2019.
